# Self-Reported Everyday Functioning After COVID-19 Infection

**DOI:** 10.1001/jamanetworkopen.2024.0869

**Published:** 2024-03-01

**Authors:** Theodore J. Iwashyna, Valerie A. Smith, Sarah Seelye, Amy S. B. Bohnert, Edward J. Boyko, Denise M. Hynes, George N. Ioannou, Matthew L. Maciejewski, Ann M. O’Hare, Elizabeth M. Viglianti, Theodore S. Berkowitz, John Pura, James Womer, Lee A. Kamphuis, Max L. Monahan, C. Barrett Bowling

**Affiliations:** 1 VA Center for Clinical Management Research, Ann Arbor VA, Ann Arbor, Michigan; 2 Department of Medicine, University of Michigan Medical School, Ann Arbor; 3 School of Medicine, Johns Hopkins University, Baltimore, Maryland; 4 School of Public Health, Johns Hopkins University, Baltimore, Maryland; 5 Center of Innovation to Accelerate Discovery and Practice Transformation, Durham VA Medical Center, Durham, North Carolina; 6 Departments of Anesthesiology, Epidemiology, and Psychiatry, University of Michigan Medical School, Ann Arbor; 7 Durham Veterans Affairs Geriatric Research Education and Clinical Center, Durham Veterans Affairs Medical Center, Durham, North Carolina; 8 Department of Medicine, Duke University, Durham, North Carolina; 9 Seattle Epidemiologic Research and Information Center, VA Puget Sound Health Care System, Seattle, Washington; 10 University of Washington, Seattle; 11 VA Puget Sound Health Care System Hospital and Specialty Medicine Service and Seattle-Denver Center of Innovation for Veteran Centered and Value Driven Care, Seattle, Washington; 12 VA Portland Healthcare System, Center to Improve Veteran Involvement in Care, Portland, Oregon; 13 College of Health, and Center for Quantitative Life Sciences, Oregon State University, Corvallis; 14 School of Nursing, Oregon Health and Science University, Portland; 15 Department of Population Health Sciences, Duke University, Durham, North Carolina

## Abstract

**Importance:**

Changes in everyday functioning are crucial to assessing the long-term impact of COVID-19 infection.

**Objective:**

To examine the impact of COVID-19 infection on everyday functioning 18 months after infection among veterans with and without histories of COVID-19 infection.

**Design, Setting, and Participants:**

This cohort study used data from the US Veterans Affairs (VA) and included 186 veterans who had COVID-19 between October 2020 and April 2021 (ie, COVID-19 cohort) and 186 matched comparators who did not have documented COVID-19 infections (ie, control cohort). This match balanced the risk of COVID-19 based on 39 variables measured in the 24 months before infection or match, using principles of target trial emulation. Data were analyzed from December 2022 to December 2023.

**Exposure:**

First documented COVID-19.

**Main Outcome and Measures:**

The differences in self-reported everyday functioning 18 months after COVID-19 infection were estimated and compared with their matched comparators. Within–matched pair logistic and linear regressions assessed differences in outcomes and were weighted to account for sampling and nonresponse.

**Results:**

Among the 186 matched pairs of participants, their weighted mean age was 60.4 (95% CI, 57.5 to 63.2) years among veterans in the COVID-19 cohort (weighted sample, 91 459 of 101 133 [90.4%] male; 30 611 [30.3%] Black or African American veterans; 65 196 [64.4%] White veterans) and 61.1 (95% CI, 57.8 to 64.4) years among their comparators in the control cohort (91 459 [90.4%] male; 24 576 [24.3%] Black or African American veterans; 70 157 [69.4%] White veterans). A high proportion of veterans in the COVID-19 cohort (weighted percentage, 44.9% [95% CI, 34.2% to 56.2%]) reported that they could do less than what they felt they could do at the beginning of 2020 compared with the control cohort (weighted percentage, 35.3%; [95% CI, 25.6% to 46.4%]; within–matched pair adjusted odds ratio [OR], 1.52 [95% CI, 0.79 to 2.91]). There was no association of documented COVID-19 infection with fatigue, substantial pain, limitations in either activities of daily living and instrumental activities of daily living, severely curtailed life-space mobility, employment, or mean health-related quality of life on a utility scale.

**Conclusions and Relevance:**

In this cohort study of veterans with and without documented COVID-19, many reported a substantial loss of everyday functioning during the pandemic regardless of whether or not they had a documented infection with COVID-19. Future work with larger samples is needed to validate the estimated associations.

## Key Points

**Question:**

Do veterans who had a history of COVID-19 report worse everyday function 18 months after their infection than veterans with a similar risk with no history of COVID-19?

**Findings:**

In this cohort study of 372 veterans, many veterans reported worse everyday function compared with how they recalled feeling before the COVID-19 pandemic. However, there was no consistent pattern of worse functioning among those who had a confirmed infection of COVID-19 compared with those without confirmed infection.

**Meaning:**

These findings suggest that the negative impacts of the COVID-19 pandemic on everyday function may occur via multiple pathways regardless of whether or not they had a documented infection with COVID-19.

## Teaser

This cohort study examines the association of COVID-19 infection with everyday functioning 18 months after infection among US veterans.

## Introduction

The World Health Organization’s clinical case definition of post–COVID-19 condition specifies that an impact on everyday functioning is essential to the diagnosis. However, many studies describing the high burden of symptoms and accumulation of new diagnoses following COVID-19 infection have failed to capture function.^1,2,3,4,5,6^ Data from general population surveys comparing respondents who had previously had COVID-19 with respondents who had not had COVID-19 may conflate the impacts of COVID-19 with factors that increased risk COVID-19 infection.^7^ Recent work extrapolating disability-adjusted life-years from new diagnoses has suggested potentially high burdens of ongoing disability attributable to COVID-19 infection,^8,9^ but did not directly measure disability.

We conceptualized everyday functioning at several levels, consistent with the international classification of functioning conceptual model^10^: (1) fatigue and pain; (2) limitations in activities of daily living; (3) limitations in social functioning (eg, life-space mobility and employment); (4) self-rated overall functioning; and (5) health-related quality of life. We measured these outcomes using a survey administered to both individuals who had previously had COVID-19 and their matched comparators. We test for differences in both the mean population and among those with the most severe symptoms.

We used target trial emulation methods to maximize rigor and compared the everyday functioning of veterans with electronic health record evidence of COVID-19 with veterans who had an equivalent risk of infection in the same month but without evidence of infection up to that point and the time of the survey (Table 1).^31^ This distinguished the impacts of the COVID-19 pandemic that may impact everyday function from specific adverse effects of documented viral infection.

**Table 1.** Target Trial Emulation Comparison Table

Element	Unethical target trial	Emulation
Goal	To test the effect of individual infection with SARS-CoV-2 on everyday functioning 18 months after infection	Same as target trial
Setting	VA nationwide system	Same as target trial
Inclusion criteria	Veterans aged 18 years and older in care in the VHA with an assigned primary care team for at least 2 years on randomization date, or who had at least 1 VHA primary care clinic visit in that period	Same as target trial
Exclusion criteria	Previous documented COVID-19 infection; address outside of Washington DC or 50 states	Previous documented SARS-CoV-2 infection in National Surveillance tool or Medicare-documented COVID-19 diagnosis or related diagnostic codes (*ICD-10-CM*: B97.29, U07.1, U09.9, J12.82, 179 Z86.16) listed in fee-for-service Medicare claims; address outside of DC or 50 States; missing or invalid key matching variables: age, height, weight, zip code; no suitable matches between infected patients and comparator
Enrollment period	October 2020 to April 2021	Same as target trial
Treatment strategies	Inoculum of SARS-CoV-2 sufficient to guarantee COVID-19 infection	SARS-CoV-2 infection with a confirmatory PCR test for SARS-CoV-2 in VA National Surveillance Tool
Comparator	Double-blinded inoculum of placebo	Best matched veteran with neither documented SARS-CoV-2 Infection in National Surveillance tool nor Medicare-documented COVID-19 diagnosis through the month at which matched as a comparator
Approach to balancing confounders	1:1 Randomization, stratified by month and center	Up to 5:1 (comparator:infected) Matching on 5 exact criteria (including month and home state) and 39 propensity score criteria from VA data
Primary outcome	Self-reported everyday functioning	Same as target trial
Follow-up period	18 Months from inoculation	18 Months from the earliest date of a documented positive test for those with COVID-19 infection; comparators began surveillance for outcomes from the same date (index date, the emulated equivalent of randomization and inoculation date) as that of their individually matched infected patient and were followed for 18 months
Causal contrast	Primary analysis: comparison of outcomes between individuals with a history of COVID-19 infection and those without contemporaneous infection	Primary analysis: same as target trial
Statistical analysis	Logistic regression for dichotomous outcomes and linear regression for continuous ones, coupled with applying inverse probability of censoring weights, compositing of death and functional outcomes, or survivor averaged causal effects to account for differential survival and loss to follow-up^31^	Within-pair differences in outcomes, using logistic or linear regression as appropriate to adjust for any unbalanced matching variables. Surveying by matched pair and enrolling a survivor in both groups eliminates differential survivorship by group; survey and nonresponse weights reweight the estimates back to the overall population of interest

Abbreviations: *ICD-10-CM*, *International Statistical Classification of Diseases, Tenth Revision, Clinical Modification*; PCR, polymerase chain reaction; VA, US Department of Veterans Affairs; VHA, Veterans Health Administration.

## Methods

This cohort study was approved by the institutional review boards (IRBs) of Ann Arbor VA and Durham VA Medical Center and uses secondary data analyses reviewed and approved by the IRBs of Durham VA Medical Center, VA Palo Alto Health Care System, VA Portland Health Care System, and VA Puget Sound Health Care System. Verbal informed consent was collected.

 We report the results of a prospective telephone- and mail-based survey collection from veterans with a history of COVID-19 and matched comparators 18 months after COVID-19 infection during October 2020 to April 2021. We focused on these patients because their onset of COVID-19 infection was late enough in the epidemic that health systems had time to adapt after the initial shock, yet health care facility-based testing was ubiquitous and home-testing was less common than now, reducing measurement error in the classification of our primary exposure.

### Study Population

As described previously,^11^ we assembled 14 separate monthly cohorts from March 2020 to April 2021 of individuals enrolled in the VA who were assigned to a VA primary care team or who had more than 1 visit to a VA primary care clinic in 2 years. Veterans included in the COVID-19 cohort were those who were first documented to be positive for COVID-19 in a given month identified via the VA COVID-19 Shared Data Resource and Medicare data. Uninfected potential comparators were those who did not have documentation of COVID-19 prior to or during the same month and met the same inclusion criteria. Comparators without a documented history of COVID-19 to date are henceforth referred to as the uninfected cohort, which is defined as no known infection. We excluded veterans with a history of COVID-19 who had COVID-19–related diagnostic codes in fee-for-service Medicare claims 15 or more days before their VA test. We convened multidisciplinary experts to select matching and control variables via a consensus-directed acyclic graph for the relationship between COVID-19 and several patient-centered outcomes.^12^ Patients with COVID-19 were exact matched with replacement to controls based on index month, sex, immunosuppressive medication use (binary), state of residence, and COVID-19 vaccination status (in January to April 2021 cohorts). Then, 39 covariates were included in month-specific propensity score models (eTable 1 in Supplement 1).^13,14,15,16^ Race and ethnicity were categorized as present in source VA data and included a mix of self-report and categorization by others. Race and ethnicity were included because of the racialization of COVID-19 exposures and access to care.

Within this broader cohort (eFigure 1 in Supplement 1), we took a stratified random sample of 100 veterans who had tested positive for COVID-19 for each month of October, November, and December 2020, and February, March, and April 2021 (eFigure 2 in Supplement 1), which was further stratified by 4 US Census regions and hospitalization. We identified up to 5 comparators for each sampled patient with COVID-19. Comparators were ranked based on how closely they were matched to a patient with COVID-19. We restricted eligible comparators to those who did not have evidence in VA or Medicare data of COVID-19 infection by April 2022, were not known to have died in VA data systems, and had an address in the US.

### Sample Size Rationale

This study was designed from 2020 to 2021 to evaluate for large mean associations. During that period, there were simultaneous discussions regarding COVID-19. For example, it was suggested that (1) COVID-19 was best understood exclusively through inpatient short-term outcomes (as in early drug trials); (2) COVID-19 might be a mass disabling event; and (3) the long-term disabling impacts of COVID-19 were independent of initial severity of illness. Thus, we designed our survey to evaluate effect sizes for activities of daily living, such as those seen in hospitalized patients with sepsis and pneumonia. For example, patients hospitalized with sepsis with no prior limitations in activities of daily living or instrumental activities of daily living reported a mean of 1.6 new limitations a year later.^17^ Similarly, hospitalizations for pneumonia were associated with 1.0 new health-related limitations in activities and instrumental activities of daily living among those with no or mild-to-moderate prehospitalization limitations.^18^

In designing the present study, we prioritized a carefully matched comparator group and population-representativeness. This required building the national sampling frame and recruiting within a complex matching structure.^11^ National representativeness as a goal meant that a veteran with COVID-19 infection needed to be recruited quickly once sampled in order to also identify and recruit a comparator without infection to retain a matched pair, which required allocating substantial resources to recruit any sampled individual. Indeed, our internal validity hinged on a high response rate for matched comparators, which we achieved.

Furthermore, the time course of evolution of late impacts of COVID-19 was unknown. As such, we designed for repeat interviews every 6 months to follow respondents, which meant that our design favored depth over breadth. Therefore, we made a pragmatic decision based on a fixed budget and at-the-time plausible large effect sizes, to attempt to survey 100 COVID-19 survivors and their matched comparators per month for each of 6 months.

There were 231 160 veterans who had a positive SARS-CoV-2 test between March 2020 and April 2021 known to VA, and 9 291 822 who did not. As previously described,^11^ 208 536 matched groups were created, and standardized mean differences (SMDs) for 39 variables between the matched groups of individuals who had previously had COVID-19 and their comparators were all less than 0.1. From the matched groups in October through December 2020 and February through April 2021, 600 potential patients with previous COVID-19 infection were identified in a stratified random sample, along with up to 5 best matched comparators (eFigure 2 in Supplement 1). Of these, 548 veterans with a history of COVID-19 infection were known to be alive and living in the US at the time of survey, and 235 (43%) consented to participate and completed surveys. Of those, 194 (83%) well-matched comparators consented and completed a survey, and 8 (4.1%) of these comparators were noted to have COVID-19 after sampling and before survey completion, resulting in 186 matched pairs.

### Survey Operations and Instruments

Approximately 18 months after the initial documented infection for COVID-19 (in monthly cohorts, April to December 2022, or to matched comparators), we sent a letter describing the survey and giving the option to opt out. Those who did not opt out were called and offered the opportunity to provide verbal informed consent for participation. Those who consented were surveyed via telephone, with an option to complete by mail or by a proxy of their choosing. Surveys could be divided throughout multiple sessions for respondents. Given the high English fluency rates among veterans of the US military, surveys were only conducted in English. A $10 token of appreciation was provided regardless of whether they completed all or part of the survey. Telephone surveys were administered by trained interviewers entering data directly into a VA Research Electronic Data Capture^19^ database with built-in validity, consistency, and completeness checks.

Once a veteran with a history of COVID-19 provided informed consent for a survey, the team attempted to recruit one of his or her 5 best matched comparators of those who were still alive and not documented to have COVID-19 per propensity score. While interviewers were aware of which cohort respondents were drawn from, they were carefully trained to administer the surveys identically and to make no reference to whether the respondent themselves had COVID-19. When asked to compare with a previous period, the respondents were to compare with the beginning of 2020, which was a time anchor relevant to all individuals.

We assembled the survey from well-validated instruments. Fatigue was assessed using the PROMIS Short Form version 1.0 Fatigue 7a and its standardized scoring,^20^ with a general population mean (SD) of 50 (10) points, with higher scores indicating more fatigue. Pain was assessed using the pain single item from the EuroQol 5-Dimension 5-Level (EQ-5D-5L). Activities of daily living and instrumental activities of daily living, were assessed using items from the Health and Retirement Study and other studies of individuals with a history of COVID-19, and tabulated as a count.^17,21,22^ Life-space mobility was assessed as in the UAB Study of Aging, with a composite score that runs from 0 to 120 with higher numbers indicating a larger, more independent life-space. EQ-5D-5L health-related quality of life scores were derived using US norms.^23^

### Statistical Analyses

Covariate balance was assessed using standardized mean differences (SMDs), where differences of less than 0.1 indicate excellent covariate balance.^16^ Associations between COVID-19 infection and survey outcomes were examined using within-pair conditional logistic regression for dichotomous outcomes and linear regression on the within-pair differences (y_COVID-19_ – y_comparator_) for continuous outcomes. All regression analyses adjusted for race and ethnicity because they were not well balanced in surveyed populations. Analyses were conducted in Stata version 17 (StataCorp) and unless otherwise noted include for both sampling weights and nonresponse weights using Stata’s probability weight adjustment procedure; R version 4.2.2 (R Project for Statistical Computing) was used to obtain the weighted SMDs for categorical variables with more than 2 categories. Stata code and log files can be found in Supplement 1 and Github.^24^

Weights incorporating both complex sampling and survey-level nonresponse were created, and the weighted results are reported here. Logistic regression was used to estimate the probability of nonresponse among eligible COVID-19 participants invited to participate. Age, sex, race, and Gagne comorbidity score were forced into the equation and other variables selected using a lasso procedure from among the variables in the matching for the month of infection. Final probability weights were the product of sampling weights and nonresponse weights.

We present analyses among individuals with a history of COVID-19 infection who were alive 18 months after onset and did not adjust for death in these estimates, as mortality analyses have been published separately.^25^ To assess robustness, we also reanalyzed the outcomes without applying weights and, separately analyzed the outcomes without removing any individuals with a history of COVID-19 prior to survey. We also conducted hypothesis-generating post hoc analyses^26,27^ separately by whether or not the COVID-19 cohort was hospitalized within 7 days of their first positive test. Statistical significance was defined as *P* < .05. Data were analyzed from December 2022 to December 2023.

## Results

Weighted characteristics of the 186 pairs included mean age of 60.4 (95% CI, 57.5-63.2) years among respondents with previous COVID-19-infection (91 459 of 101 133 in the weighted sample [90.4%] male; 30 611 [30.3%] Black or African American veterans; 65 196 [64.4%] White veterans; 40 721 [40.3%] had never smoked; 11 194 [11.1%] had been prescribed immunosuppressant medicines in the previous 24 months) and 61.1 (95% CI, 57.8-64.4) years among their comparators (91 459 [90.4%] male; 24 576 [24.3%] Black or African American veterans; 70 157 [69.4%] White veterans; 37 956 [37.5%] had never smoked; 11 194 [11.1%] had been prescribed immunosuppressant medicines by VA in previous 24 months) (Table 2).

**Table 2.** Weighted Descriptive Statistics for COVID-19 Cases and Their Matched Comparators^a^

Characteristics	Participants, mean (95% CI)		SMD
	COVID-19 cases (unweighted n = 186; weighted n = 101 132)	Matched uninfected comparators (unweighted n = 186; weighted n = 101 132)	
Age, y	60.4 (57.5-63.2)	61.1 (57.8-64.4)	0.05
Sex, No. (%)			
Female	9619 (9.5)	9619 (9.5)	0
Male	91 459 (90.4)	91 459 (90.4)	
Unknown	55 (0.1)	55 (0.1)	
Race, No. (%)			
Black or African American	30 611 (30.3)	24 576 (24.3)	0.14
White	65 196 (64.4)	70 157 (69.4)	
Other^b^	5326 (5.3)	6399 (6.3)	
Hispanic ethnicity, No. (%)			
Yes	3280 (3.2)	8399 (8.3)	0.22
No	97 852 (96.8)	92 733 (91.7)	
Missing			
Rurality, No. (%)			
Urban	73 970 (73.1)	63 443 (62.7)	0.22
Not urban (incl. missing)	27 162 (26.9)	37 689 (37.3)	
Smoking Status, No. (%)			
Current	9438 (9.3)	12 399 (12.3)	0.21
Former	47 616 (47.1)	43 325 (42.8)	
Never	40 721 (40.3)	37 956 (37.5)	
Missing	3358 (3.3)	7452 (7.4)	
Gagne score	1.3 (0.9-1.7)	1.1 (0.6-1.6)	0.10
No. of previous 24 mo VA inpatient admissions, mean (CI)	33.7 (11.2-5.1)	44.1 (15.1-73.1)	0.09
No. of previous 24 mo VA primary care visits	10.5 (8.1-13.0)	10.7 (8.1-13.4)	0.02
No. of previous 24 mo VA specialty care visits	15.9 (12.4-19.4)	17.0 (12.5-21.5)	0.06
No. of previous 24 mo VA mental health care visits	7.5 (3.6-11.5)	3.7 (2.2-5.2)	0.27
Immunosuppressed in prior 24 mo, No. (%)	11 194 (11.1)	11 194 (11.1)	0
Community living center (VA nursing home) at index date, No. (%)	0	0	0
NOSOS score	1.2 (1.0-1.4)	1.2 (1.0-1.4)	0.007
CAN Score	57.6 (51.2-64.0)	55.8 (49.6-62.0)	0.07
Distance to nearest VAMC, miles	34.0 (25.9-42.0)	37.2 (29.5-45.0)	0.09

Abbreviations: CAN, Care Assessment Need score; SMD, standardized mean difference; VA, Veterans Affairs; VAMC, Veterans Affairs Medical Center.

^a^ Unweighted data can be found in eTable 2 in Supplement 1.

^b^ The other racial category includes those identified in the VA source data as American Indian or Alaska Native, Asian, Native Hawaiian or Other Pacific Islander, multiple races, or for whom the data were missing.

### Fatigue and Pain

Fatigue scores reported 18 months after COVID-19 infection had a mean of 54.6 (95% CI, 52.6-56.7), where higher is worse. Rates of fatigue were similar in the comparator group, with a mean fatigue score of 51.7 (95% CI, 49.5 to 53.9). The within–matched pair adjusted regression showed no statistically significant association between COVID-19 infection and fatigue score (Table 3). Of veterans in the COVID-19 cohort, 51.1% (95% CI, 40.1% to 62.0%) reported moderate, severe, or extreme pain at 18 months vs 65.7% (95% CI, 54.7% to 75.3%) of their comparators. The within–matched pair conditional logistic regression showed no statistically significant difference in odds of reporting substantial pain by group (odds ratio [OR], 0.50 [95% CI, 0.24 to 1.05]) (Table 4).

**Table 3.** COVID-19 and Thresholds of Morbidity at 18 Months^a^

Characteristic	Weighted association with COVID-19, OR (95% CI)
Pain (moderate, severe, extreme limitation)	0.50 (0.24-1.05)
Severe (4 or more) I/ADL limitation	1.46 (0.75-2.84)
Curtailed life space (<60)	0.90 (0.44-1.82)
Not employed	0.89 (0.45-1.77)
Poor health-related quality of life, EQ-5D-5L<0.5)	1.37 (0.64-2.91)
At less than 75% of 2020 functioning	1.52 (0.79-2.91)

Abbreviations: EQ-5D, EuroQol 5-Dimension 5-Level; I/ADL, activities and instrumental activities of daily living; OR, odds ratio.

^a^ Weighted within-pair conditional logistic regression, adjusted for race and ethnicity. Confidence intervals including 1 indicate no statistically significant association; odds ratios greater than 1 indicate more common after COVID-19 than in matched comparators.

**Table 4.** COVID-19 With Continuous Measures of Morbidity at 18 Months^a^

Measure	Interpretation of direction of coefficient	Weighted association with COVID-19 coefficient (95% CI)
Fatigue score	Higher is more fatigued (score ranges 0-1)	0.33 (−3.19 to 3.85)
I/ADL limitations count	Higher is more limitations (score ranges 0-13)	−0.04 (−1.24 to 1.15)
Composite life space score	Lower is more curtailed life space (score ranges 0-120)	8.89 (−3.90 to 21.69)
EQ-5D utility index	Lower is worse health-related quality of life (score ranges 0-1)	−0.02 (−0.14 to 0.10)
Extent to which respondent feels back to January 2020 functioning	Lower is worse functioning (score ranges 0-100)	−5.22 (−14.65 to 4.21)

Abbreviations: EQ-5D, EuroQol 5-Dimension 5-Level; I/ADL, activities of daily living or instrumental activities of daily living.

^a^ Weighted paired regression, adjusted for race and ethnicity. Coefficients represent the absolute increase (decrease if negative) in the mean scores of COVID-19 infected patients compared with their specific matched comparator. Confidence intervals including 0 indicate no statistically significant association; positive coefficients indicate more common after COVID-19 than in matched comparators.

### Activities of Daily Living

The distribution of health-related limitations in activities of daily living and instrumental activities of daily living at 18 months after COVID-19 infection is the Figure. Veterans in the COVID-19 cohort reported a mean of 3.4 (95% CI, 2.6 to 4.2) limitations, and 37.3% (95% CI, 27.5% to 48.3%) reported 4 or more activities of daily living or instrumental activities of daily living limitations. Matched comparators reported a mean of 3.0 (95% CI, 2.4 to 3.6) limitations, and 30.2% (95% CI, 21.3% to 40.9%) reported 4 or more limitations. There was no difference in the within-pair analysis of the mean (SMD, −0.04 [95% CI, −1.24 to 1.15] fewer limitations in COVID-19 cohort), nor in the odds of reporting 4 or more ADL or IADL limitations (OR, 1.46 [95% CI, 0.75 to 2.84]).

**Figure.**
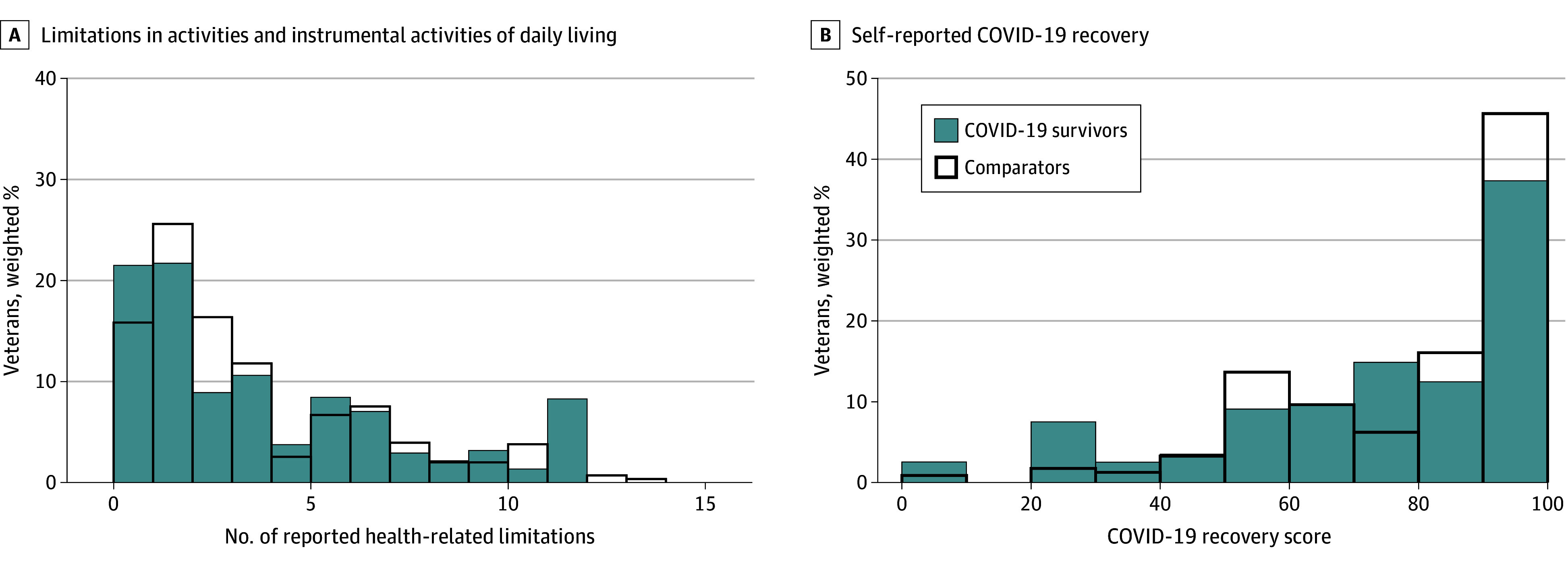
Distributions of Self-Reported Limitations in Activities and Instrumental Activities of Daily Living and Answers Regarding Physical and Mental Ability Since 2020 Weighted results are presented.

### Life-Space Mobility and Employment

Life-space mobility at 18 months after infection was similar for the COVID-19 cohort and their matched comparators (mean [SD], 70.4 [33.1] vs 66.6 [28.3], where higher is better; within–matched pair adjusted regression nonsignificant difference of 8.89 points better life-space mobility after COVID-19, 95% CI, −3.90 to 21.69). There was also a similar concentration of individuals with severely curtailed life-space mobility among both cohorts, with 37.0% (95% CI, 27.1% to 48.1%) of the COVID-19 cohort and 41.1% (95% CI, 30.6% to 52.4%) of the control cohort having scores less than 60 (within–matched pair adjusted conditional logistic regression OR, 0.90; 95% CI, 0.44 to 1.82). Overall, 62.6% (95% CI, 51.7% to 72.4%) of COVID-19 cohort was not employed at the 18-month follow-up compared with 64.7% (95% CI, 53.7% to 74.3%) of their comparators. The OR from within-pair adjusted regression was 0.89 (95% CI, 0.45 to 1.77).

### Quality of Life

Respondents were asked “Think about what you could do physically and mentally at the beginning of the year 2020. On a scale of 1 to 100, with 100 being all the way back to what you could do at the start of 2020, how close to being back are you?” Of the veterans in the COVID-19 cohort, 44.9% (95% CI, 34.2% to 56.2%) reported they could do less than 75% of what they felt they could do at the beginning of 2020, in contrast with 35.3% (95% CI, 25.6% to 46.4%) of their comparators (Figure). The within–matched pair adjusted OR was 1.52 (95% CI, 0.79 to 2.91). Mean (SD) EQ-5D-5L health utility scales at 18 months were 0.57 (0.35) in the COVID-19 cohort and 0.61 (0.25) in the comparators, with a within–matched pair adjusted difference of −0.02 (95% CI, −0.14 to 0.10).

### Sensitivity Analyses

eTables 3 to 6 in Supplement 1 shows similar results in unweighted analyses and in weighted analyses that did not exclude the 8 comparators ascertained from medical records to have COVID-19 after sampling but prior to survey completion. Hypothesis-generating post hoc analyses^26,27^ shown in eTables 7 and 8 suggest possible enduring impacts of COVID-19 among the hospitalized group on fatigue and function, 18 months later, but standard errors were large. eTable 9 in Supplement 1 examines health care use among survey respondents with documented COVID-19 compared with those sampled but who did not participate in the survey and shows somewhat greater rates of primary care interaction by survey respondents, but no statistically significant differences in inpatient admission rates, specialty care interactions, or mental health interactions.

## Discussion

Our results suggested a high burden of ongoing fatigue, pain, and disability among veterans after the COVID-19 pandemic. When asked in 2022, they rated their own physical and mental functioning as substantially worse than it was in 2020. Our sample, as finally collected, was too small to provide precise evidence as to whether or not this morbidity was more common among those with documented COVID-19 infection than among those who without.

This reduced sample size provides an important nuance to interpreting the lack of a statistically significant association. Consider our results for activities of daily living. We found an effect size of potential interest (OR, 1.46) in the association of COVID-19 and having 4 or more activities of daily living or instrumental activities of daily living limitations, but the confidence interval was wide (95% CI, 0.75 to 2.84). This means that our data cannot reject the null hypothesis that the rates of substantial activities of daily living limitation are the same in among surviving individuals in the COVID-19 cohort and their risk-matched comparators. However, our data are also consistent with individuals with a history of COVID-19 having double the odds of substantial disability—the sample we were able to obtain may have lacked the power to distinguish these quite different scenarios. Our results do establish bounds such that effect sizes as large as the mean increase in activities of daily living or instrumental activities of daily living limitations seen in inpatient sepsis^17^ are inconsistent with our data.

In the face of lack of power, there is sometimes a temptation to seek signal in point estimates, or in consistency of direction across multiple outcome measures. Our data suggest the limits of such a strategy for providing strong, reproducible evidence. The point estimates were inconsistent across different tests of association in the same measure. For example, the potentially large OR of 1.46 for more than 4 limitations coexists with a mean difference of −0.04 fewer activities of daily living or instrumental activities of daily living limitations in the COVID-19 cohort, with a broad 95% CI. We did not possess strong a priori reasons for preferring one approach to testing for a difference associated with COVID-19.

These data have advantages and weaknesses. Unlike some assessments of COVID-19, we use self-reported data for the infection and control groups. Doing so in our sampling and within–matched pairs analysis controls for the unequal distribution of COVID-19 among the population, which evolved throughout the first years of the epidemic. Our use of self-report with the same questions allowed direct comparisons of the results between matched pairs. We reduced bias by using a national sampling frame and weighting to incorporate sampling and nonresponse. The baseline burden of morbidity among VA patients may limit the ability to detect disease impacts^28^ and generalizability to other populations. This combination of features are uncommon among the more than 196 studies summarized in recent major publications and systematic reviews.^1,2,3,4,5,6^ Our results contrast with studies extrapolating disability-adjusted life-years from new diagnoses attributed to COVID-19 infection,^8,9^ but that do not directly measure disability. Differential ascertainment may be a greater risk for claims-based studies, and our study attempted to minimize this.

These data are consistent with an interpretation that the COVID-19 pandemic has had adverse effects outside of solely infectious or postinfectious mechanisms. Other adverse effects may have been due to psychological, behavioral, social, policy, and economic mechanisms. These data cannot rule out the possibility that COVID-19–confirmed viral infection may be associated with disability among some individuals who had COVID-19.^29^ We value the assessments of respondents themselves, in which they self-reported decline in physical and mental capacity relative to 2020.

### Limitations

This study has limitations. We prioritized self-report rather than physiological or objective functional testing, which could introduce recall bias in cases where veterans were asked to compare with their own recalled prior capacity. The age and sex distribution of the sample reflect the VA, which may not be generalizable across the whole population.^30^

Because our data were collected via survey, we did not include individuals who had died, which may violate target trial emulation conditioned on postrandomization information.^27^ We have previously measured that the population-weighted differences in mortality,^25^ which could introduce censoring of the extreme phenotype bias.^31^ Our study was further limited by the small sample size, which was partially due to a lower-than-expected recruitment rate, which has been seen in other studies.^32,33,34,35^ Future work should include larger sample sizes to better estimate the association of COVID-19 with everyday functioning.

We sampled too few female or nonbinary respondents to explore sex and gender differences. Occupation information was also not available.^36^ Although an acyclic directed graph-informed target trial emulation was used to maximize rigor,^12^ unmeasured confounding could persist. The degree of undocumented infection among comparators is unknown.

## Conclusions

In this cohort study, veterans reported high rates of ongoing fatigue, pain, and disability after the COVID-19 pandemic, regardless of history of COVID-19. There were no statistically significant results between these symptoms and COVID-19 illness. These results highlight the importance of acknowledging and addressing the broader impacts of the COVID-19 pandemic on health beyond those directly associated with documented infection.

## Notes

Supplement 1. **eTable 1.** Variables Included in Propensity Score **eTable 2.** Descriptive Statistics and Standardized Mean Differences for COVID-19 Cases and Their Matched Comparators **eTable 3.** Association of COVID-19 With Thresholds of Morbidity at 18 Months **eTable 4.** Association of COVID-19 With Continuous Measures of Morbidity at 18 Months **eTable 5.** Association of COVID-19 With Thresholds of Morbidity at 18 Months **eTable 6.** Association of COVID-19 With Continuous Measures of Morbidity at 18 Months **eTable 7.** Association of COVID-19 With Thresholds of Morbidity at 18 Months **eTable 8.** Association of COVID-19 With Continuous Measures of Morbidity at 18 Months **eTable 9.** Association of Survey Participation With Post-COVID-19 VA Healthcare Utilization, Among Those With Documented COVID-19 **eFigure 1.** Study Flow Diagram for Overall Cohort **eFigure 2.** Study Flow Diagram for COVID-19 Infected Respondents

Supplement 2.
Nonauthor Collaborators

Supplement 3.
Data Sharing Statement
