# Supplementary material for: Self-Reported Everyday Functioning After COVID-19 Infection
Source: JAMA Netw Open. 2024 Mar 1;7(3):e240869. doi: 10.1001/jamanetworkopen.2024.0869 (PMC10907923; doi:10.1001/jamanetworkopen.2024.0869)
Supplement: Supplement 1. — eTable 1. Variables Included in Propensity Score eTable 2. Descriptive Statistics and Standardized Mean Differences for COVID-19 Cases and Their Matched Comparators eTable 3. Association of COVID-19 With Thresholds of Morbidity at 18 Months eTable 4. Association of COVID-19 With Continuous Measures of Morbidity at 18 Months eTable 5. Association of COVID-19 With Thresholds of Morbidity at 18 Months eTable 6. Association of COVID-19 With Continuous Measures of Morbidity at 18 Months eTable 7. Association of COVID-19 With Thresholds of Morbidity at 18 Months eTable 8. Association of COVID-19 With Continuous Measures of Morbidity at 18 Months eTable 9. Association of Survey Participation With Post-COVID-19 VA Healthcare Utilization, Among Those With Documented COVID-19 eFigure 1. Study Flow Diagram for Overall Cohort eFigure 2. Study Flow Diagram for COVID-19 Infected Respondents [file jamanetwopen-e240869-s001.pdf]

Supplemental Online Content

Iwashyna TJ, Smith VA, Seelye S, et al. Self-reported everyday function after COVID-19 infection. *JAMA Netw*. 2024;7(3):e240869. doi:10.1001/jamanetworkopen.2024.0869

eTable 1. Variables Included in Propensity Score
eTable 2. Descriptive Statistics and Standardized Mean Differences for COVID-19 Cases and Their Matched Comparators
eTable 3. Association of COVID-19 With Thresholds of Morbidity at 18 Months
eTable 4. Association of COVID-19 With Continuous Measures of Morbidity at 18 Months
eTable 5. Association of COVID-19 With Thresholds of Morbidity at 18 Months
eTable 6. Association of COVID-19 With Continuous Measures of Morbidity at 18 Months
eTable 7. Association of COVID-19 With Thresholds of Morbidity at 18 Months
eTable 8. Association of COVID-19 With Continuous Measures of Morbidity at 18 Months
eTable 9. Association of Survey Participation With Post-COVID VA Healthcare Utilization, Among Those With Documented COVID-19
eFigure 1. Study Flow Diagram for Overall Cohort
eFigure 2. Study Flow Diagram for COVID-19 Infected Respondents

This supplemental material has been provided by the authors to give readers additional information about their work.

© 2024 Iwashyna TJ et al. *JAMA Netw Open.*

eTable 1: Variables included in propensity score

Construction of all variables and matches have been previously described at https://bmcmedresmethodol.biomedcentral.com/articles/10.1186/s12874-023-01882-z

Categorical variables included in propensity score:

- immunosuppressive medication use
- nursing home residence any time
- sex
- race/ethnicity
- rurality
- state of residence
- smoking status
- categorization of two comorbidity scores (CAN[12], Nosos[13])
- Indicators for diagnosed CDC high-risk conditions based on ICD-19 codes: coronary heart disease, cancer (excluding non-metastatic skin cancers), chronic kidney disease, congestive heart failure, pulmonary-associated conditions (including asthma, COPD, interstitial lung disease, and cystic fibrosis), dementia, diabetes, hypertension, liver disease, sickle cell/thalassemia, solid organ or blood stem cell transplant, stroke/cerebrovascular disorders, substance use disorder, anxiety disorder, bipolar disorder, major depression, PTSD, and schizophrenia
- Vaccination status (January-April 2021)

Continuous covariates included:

- age
- body mass index (BMI)
- Gagne comorbidity score
- distance from a Veteran's home to nearest VA hospital
- count of CDC high-risk conditions
- count of mental health conditions
- four VA utilization measures (inpatient admissions, primary care visits, specialty care visits, mental health visits in the prior 2 years).

© 2024 Iwashyna TJ et al. *JAMA Netw Open.*

eTable 2: Descriptive Statistics and Standardized Mean Differences for COVID-19 Cases and Their Matched Comparators *(unweighted)*

	**COVID-19 Cases** (N=186)	**Comparators** (N=186)	**SMD**
Age, years, mean (SD)	59.3 (14.2)	58.9 (14.5)	0.030
BMI, mean (SD)	31.9 (6.2)	32.2 (6.8)	0.041
Sex, N (%)			
Female	22 (11.8)	22 (11.8)	
Male	163 (87.6)	163 (87.6)	0
Unknown	1 (0.5)	1 (0.5)	
Race, N (%)			
Black or African American	46 (24.7)	47 (25.3)	
White	128 (68.8)	116 (62.4)	0.210
Other	12 (6.5)	23 (12.4)	
Hispanic Ethnicity, N (%)			
Yes	12 (6.5)	19 (10.2)	
No	174 (93.5)	167 (89.8)	0.136
Missing			
Rurality, N (%)			
Urban	130 (69.9)	124 (66.7)	0.069
Not Urban (incl. missing)	56 (30.1)	62 (33.3)	
Smoking Status, N (%)			
Current	17 (9.1)	24 (12.9)	
Former	78 (41.9)	73 (39.2)	0.126
Never	83 (44.6)	80 (43.0)	
Missing	8 (4.3)	9 (4.8)	
Gagne score, mean (SD)	1.1 (1.8)	0.9 (1.9)	0.104
# Previous 24 Month VA Inpatient Admissions, mean (SD)	0.3 (0.9)	0.2 (0.9)	0.012
# Previous 24 Month VA Primary Care Visits, mean (SD)	9.6 (9.6)	8.7 (9.7)	0.090
# Previous 24 Month VA Specialty Care Visits, mean (SD)	15.1 (14.3)	14.2 (15.5)	0.061
Previous 24 Month Mental Health Care Utilization, mean (SD)	8.2 (18.1)	6.8 (17.4)	0.079
Immunosuppressed in prior 24 months, N (%)	15 (8.1)	15 (8.1)	0
Community Living Center (VA Nursing Home) at Index Date, N (%)	0	0	
NOSOS score, mean (SD)	1.2 (1.0)	1.2 (0.9)	0.006
CAN Score, mean (SD)	56.0 (29.1)	53.3 (27.4)	0.096
Distance to nearest VAMC (miles)	36.3 (36.9)	34.5 (31.2)	0.054

© 2024 Iwashyna TJ et al. *JAMA Netw Open.*

eTable 3. Association of COVID-19 with Thresholds of Morbidity at 18 months.
Unweighted within-pair conditional logistic regression, adjusted for race and ethnicity. Confidence intervals including 1 indicate no statistically significant association; odds ratios greater than 1 indicate more common after COVID-19 than in matched comparators. I/ADL: activities and instrumental activities of daily living.

	**Unweighted Association with COVID-19**	
	**Odds Ratio**	**95% Confidence Interval**
Pain (moderate, severe, extreme limitation)	0.98	0.62, 1.55
Severe (4 or more) I/ADL limitation	1.35	0.87, 2.11
Curtailed life space (<60)	0.86	0.53, 1.38
Not Employed	0.81	0.51, 1.29
Poor health-related quality of life, EQ-5D-5L <0.5)	1.23	0.74, 2.05
At less than 75% of 2020 functioning	1.22	0.76, 1.95

© 2024 Iwashyna TJ et al. *JAMA Netw Open.*

eTable 4. Association of COVID-19 with Continuous Measures of Morbidity at 18 months. Unweighted paired regression, adjusted for race and ethnicity. Coefficients represent the absolute increase (decrease if negative) in the average scores of COVID-19 infected patients compared to their specific matched comparator. Confidence intervals including 0 indicate no statistically significant association; positive coefficients indicate more common after COVID-19 than in matched comparators. I/ADL: activities and instrumental activities of daily living.

	**Interpretation of Direction of Coefficient**	**Unweighted Association with COVID-19**	
		**Coeff-icient**	**95% Confidence Interval**
Fatigue score	Higher is more fatigued	0.73	-1.46, 2.92
I/ADL limitations count	Higher is more limitations	-0.07	-0.85, 0.71
Composite life space score	Lower is more curtailed life space	12.23	4.68, 19.77
EQ-5D utility index	Lower is worse health-related quality of life	-0.02	-0.09, 0.06
Extent to which respondent feels back to January 2020 functioning	Lower is worse functioning	-5.00	-11.00, 1.00

© 2024 Iwashyna TJ et al. *JAMA Netw Open.*

eTable 5. Association of COVID-19 with Thresholds of Morbidity at 18 months. Weighted within-pair conditional logistic regression, adjusted for race and ethnicity, including 8 pairs where the comparator was later found to have COVID-19. Confidence intervals including 1 indicate no statistically significant association; odds ratios greater than 1 indicate more common after COVID-19 than in matched comparators. I/ADL: activities and instrumental activities of daily living.

Including 8 pairs where the comparator was later found to have COVID-19	**Weighted Association with COVID-19**	
	**Odds Ratio**	**95% Confidence Interval**
Pain (moderate, severe, extreme limitation)	0.60	0.30, 1.20
Severe (4 or more) I/ADL limitation	1.47	0.76, 2.87
Curtailed life space (<60)	0.94	0.49, 1.82
Not Employed	0.86	0.44, 1.66
Poor health-related quality of life, EQ-5D-5L <0.5)	1.26	0.61, 2.63
At less than 75% of 2020 functioning	1.52	0.80, 2.87

© 2024 Iwashyna TJ et al. *JAMA Netw Open.*

eTable 6. Association of COVID-19 with Continuous Measures of Morbidity at 18 months. Weighted paired regression, adjusted for race and ethnicity, including 8 pairs where the comparator was later found to have COVID-19. Coefficients represent the absolute increase (decrease if negative) in the average scores of COVID-19 infected patients compared to their specific matched comparator. Confidence intervals including 0 indicate no statistically significant association; positive coefficients indicate more common after COVID-19 than in matched comparators. I/ADL: activities and instrumental activities of daily living.

Including 8 pairs where the comparator was later found to have COVID-19	**Interpretation of Direction of Coefficient**	**Weighted Association with COVID-19**	
		**Coeff-icient**	**95% Confidence Interval**
Fatigue score	Higher is more fatigued	0.40	-3.00, 3.80
I/ADL limitations count	Higher is more limitations	0.01	-1.14, 1.17
Composite life space score	Lower is more curtailed life space	7.96	-4.46, 20.37
EQ-5D utility index	Lower is worse health-related quality of life	-0.02	-0.13, 0.10
Extent to which respondent feels back to January 2020 functioning	Lower is worse functioning	-5.82	-15.09, 3.45

© 2024 Iwashyna TJ et al. *JAMA Netw Open.*

eTable 7. Association of COVID-19 with Thresholds of Morbidity at 18 months (Analyzed Separately by Whether Initial COVID-19 Case was Hospitalized or Not). Weighted within-pair conditional logistic regression, adjusted for race and ethnicity. Confidence intervals including 1 indicate no statistically significant association; odds ratios greater than 1 indicate more common after COVID-19 than in matched comparators. 32 COVID-19 patients were hospitalized within 7-days of their initial positive test in VA. Note that these should be considered hypothesis generating in a target trial emulation framework, as whether or not COVID-19 leads to an acute hospitalization may be part of the causal pathway by which COVID-19 causes adverse mortality. In the language of clinical trials that target trial emulation seeks to follow, hospitalization is a "post-randomization" variable.

I/ADL: activities and instrumental activities of daily living.

	**Weighted Association with COVID-19 among Hospitalized Cases**		**Weighted Association with COVID-19 among Non-Hospitalized Cases**	
	**Odds Ratio**	**95% Confidence Interval**	**Odds Ratio**	**95% Confidence Interval**
Pain (moderate, severe, extreme limitation)	0.21	0.05, 0.91	0.56	0.24, 1.28
Severe (4 or more) I/ADL limitation	1.96	0.51, 7.50	1.48	0.68, 3.24
Curtailed life space (<60)	0.43	0.08, 2.41	0.94	0.42, 2.10
Not Employed	0.64	0.10, 4.16	0.89	0.43, 1.84
Poor health-related quality of life, EQ-5D-5L <0.5)	1.13	0.29, 4.45	1.44	0.57, 3.64
At less than 75% of 2020 functioning	6.30	0.76, 52.36	1.33	0.64, 2.76

© 2024 Iwashyna TJ et al. *JAMA Netw Open.*

eTable 8. Association of COVID-19 with Continuous Measures of Morbidity at 18 months (Analyzed Separately by Whether Initial COVID-19 Case was Hospitalized or Not). Weighted paired regression, adjusted for race and ethnicity. Coefficients represent the absolute increase (decrease if negative) in the average scores of COVID-19 infected patients compared to their specific matched comparator. Confidence intervals including 0 indicate no statistically significant association; positive coefficients indicate more common after COVID-19 than in matched comparators. 32 COVID-19 patients were hospitalized within 7-days of their initial positive test in VA. Note that these should be considered hypothesis generating in a target trial emulation framework, as whether or not COVID-19 leads to an acute hospitalization may be part of the causal pathway by which COVID-19 causes adverse mortality. In the language of clinical trials that target trial emulation seeks to follow, hospitalization is a "post-randomization" variable.

I/ADL: activities and instrumental activities of daily living.

	**Weighted Association with COVID-19 among Hospitalized Cases**		**Weighted Association with COVID-19 among non-Hospitalized Cases**	
	**Coeff-icient**	**95% Confidence Interval**	**Coeff-icient**	**95% Confidence Interval**
Fatigue score	5.31	1.98, 8.65	-0.42	-4.42, 3.58
I/ADL limitations count	1.16	-0.82, 3.15	-0.25	-1.60, 1.11
Composite life space score	13.54	-8.92, 35.99	8.51	-5.88, 22.89
EQ-5D utility index	-0.07	-0.30, 0.15	-0.005	-0.14, 0.13
Extent to which respondent feels back to January 2020 functioning	-14.27	-31.30, 2.77	-4.00	-14.60, 6.59

© 2024 Iwashyna TJ et al. *JAMA Netw Open.*

eTable 9. Association of Survey Participation with Post-COVID VA Healthcare Utilization, Among those with Documented COVID-19. Unweighted regression. We sought to assess the extent to which survey respondents were systematically different than non-respondents in their experience of COVID-19. Differences in pre-COVID-19 characteristics, we reasoned, would be rebalanced based on our non-response and other survey weights. So we examined observed differences in health care utilization among survey respondents who had documented COVID-19 (and consented to linkage to their medical records, n=164), compared to those Veterans eligible for the survey but who were not successfully contacted or did not offer consent to the survey (n=341). We used the month-by-month data on utilization constructed as part of the inverse probability of censoring weights for a related project in this cohort and for which methods have been published (PMID: 37603339).

We examined 5 measures: inpatient admissions (dichotomous); and number of primary care interactions (continuous); mental health interactions (continuous); and of specialty care interactions. We attempted to examine community-living center admission (dichotomous), but it was too rare in this cohort to estimate. We adjusted in these analyses for all the same variables included in the LASSO regression in this paper to build the non-response and survey weights. There were no significant differences in rates of admission, specialty care interactions, or mental health interactions, but survey responders did have modestly more primary care interactions. We interpret this cautiously as suggesting broadly similar health experiences between respondents and non-respondents, but with somewhat greater comfort among survey respondents in interacting with the VA healthcare system.

Among those with documented COVID-19	**Adjusted Difference Between Responders And Non-Responders**
	Odds Ratio (95% CI)
Inpatient admission	0.54 (0.16, 1.84)
	Coef. (*p*-value)
# primary care interactions	3.46 (0.003)
# specialty care interactions	0.81 (0.364)
# mental health care interactions	0.83 (0.319)

© 2024 Iwashyna TJ et al. *JAMA Netw Open.*

eFigure 1: Study Flow Diagram for Overall Cohort, as previously described at https://bmcmedresmethodol.biomedcentral.com/articles/10.1186/s12874-023-01882-z

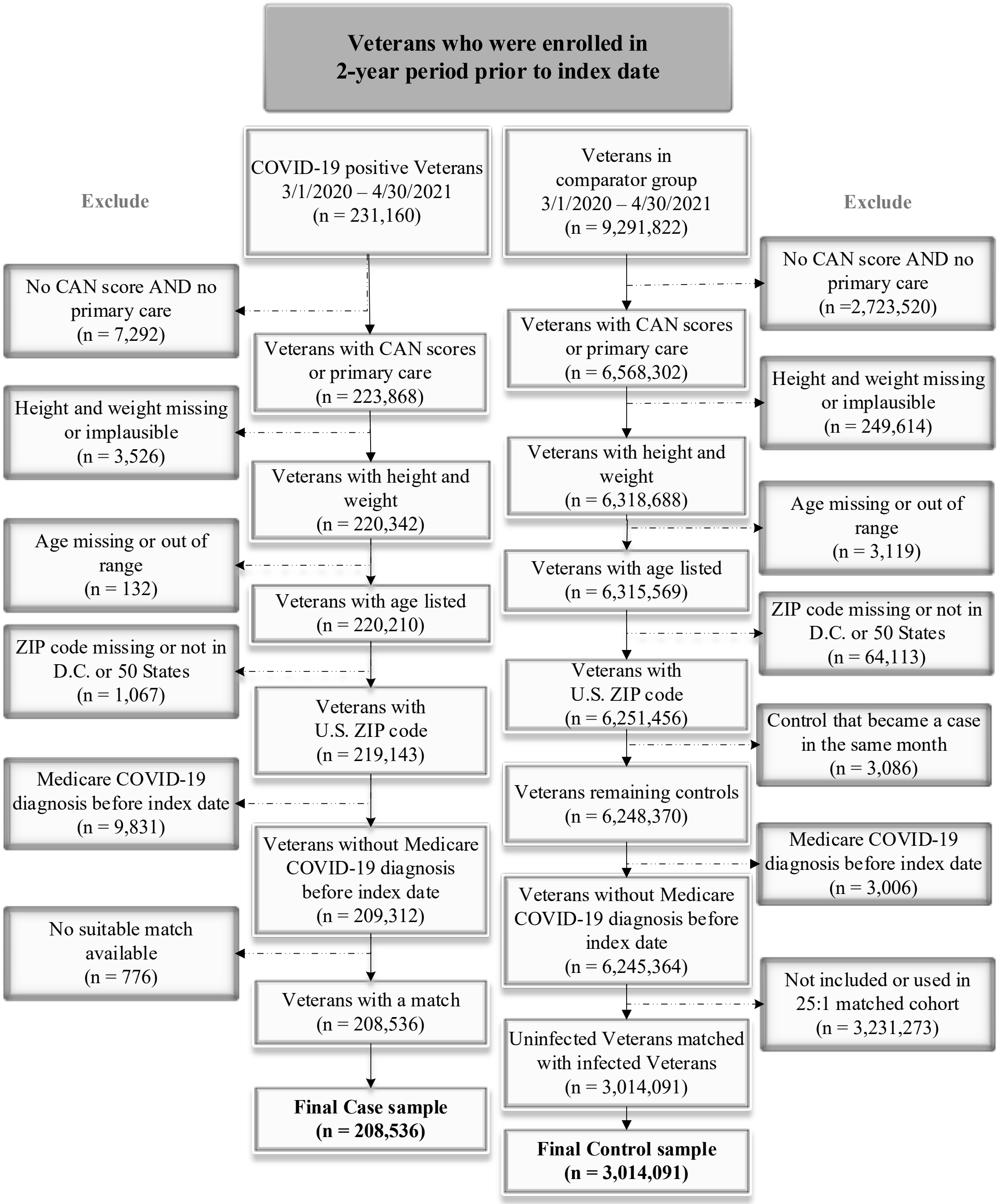

© 2024 Iwashyna TJ et al. *JAMA Netw Open.*

eFigure 2: Study Flow Diagram for COVID-19 Infected Respondents

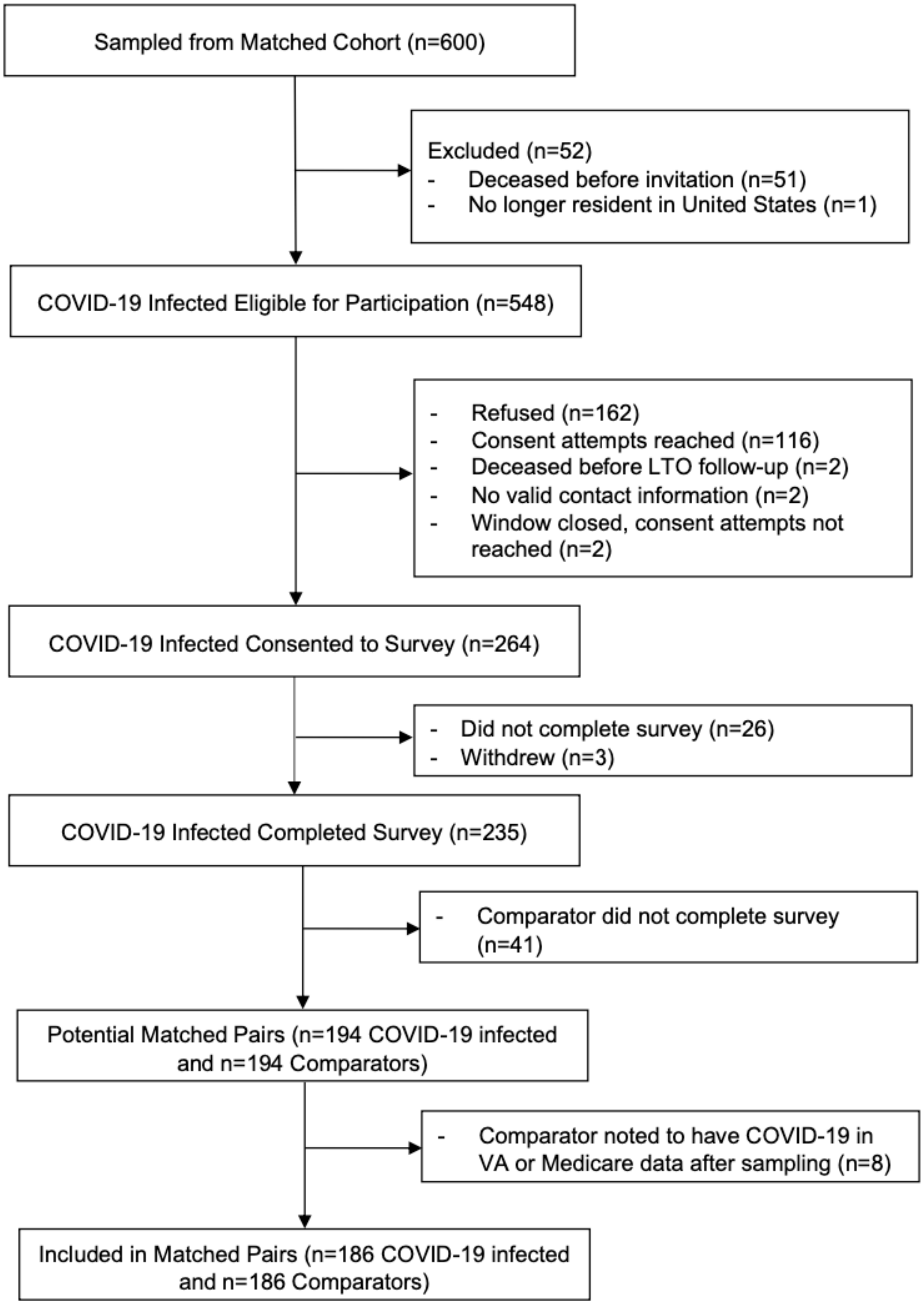

© 2024 Iwashyna TJ et al. *JAMA Netw Open.*
