# Supplementary material for: Self-Reported Everyday Functioning After COVID-19 Infection
Source: JAMA Netw Open. 2024 Mar 1;7(3):e240869. doi: 10.1001/jamanetworkopen.2024.0869 (PMC10907923; doi:10.1001/jamanetworkopen.2024.0869)
Supplement: Supplement 2. — Nonauthor Collaborators [file jamanetwopen-e240869-s002.pdf]

Supplemental Online Content: Nonauthor Collaborators
*First name, last name, and suffix (if applicable) are required and will appear in PubMed.

*Group Name(s): VA HSR&D COVID-19 Observational Research Collaboratory (CORC)							
***First Name and Middle Initial(s)**	***Last Name**	***Suffix (eg, Jr, III)**	Academic Degrees	Institution	Location (city, state/province, country)	Role or Contribution, eg, chair, principal investigator	Group (if more than 1 Group listed in the byline) and/or Subgroup (eg, Steering Committee)
Andrew	Admon		MD	VA Ann Arbor Healthcare System	Ann Arbor, MI	Site Collaborator	CORC Participating Collaborators
Kathleen	Akgun		MD	VA Connecticut Healthcare System	West Haven, CT	Site Collaborator	CORC Participating Collaborators
Stacy	Anderson		BA	VA Ann Arbor Healthcare System	Ann Arbor, MI	Research Assistant	CORC Participating Collaborators
Mihaela	Aslan		PhD	VA Connecticut Healthcare System	West Haven, CT	Site Collaborator	CORC Participating Collaborators
David	Au		MD	VA Puget Sound Healthcare System	Seattle, WA	Physician Consultant	CORC Participating Collaborators
Lisa	Backus		MD	VA Palo Alto Healthcare System	Palo Alto, CA	Site Collaborator	CORC Participating Collaborators
Kristina	Bajema		MD	VA Portland Healthcare System	Portland, OR	Site Collaborator	CORC Participating Collaborators
Aaron	Baraff		MS	VA Puget Sound Healthcare System	Seattle, WA	Data Analyst	CORC Participating Collaborators
Lisa	Batten		MD	VA Puget Sound Healthcare System	Seattle, WA	Project Manager	CORC Participating Collaborators
Theodore	Berkowitz		MS	VA Durham Healthcare System	Durham, NC	Data Manager	CORC Participating Collaborators
Taylor	Bernstein		MPH	VA Ann Arbor Healthcare System	Ann Arbor, MI	Research Assistant	CORC Participating Collaborators
Kristin	Berry Wyatt		PhD	VA Puget Sound Healthcare System	Seattle, WA	Data Manager	CORC Participating Collaborators
Joseph	Bogdan		BA	VA Durham Healthcare System	Durham, NC	Project Manager	CORC Participating Collaborators
Hayden	Bosworth		PhD	VA Durham Healthcare System	Durham, NC	Site Collaborator	CORC Participating Collaborators

Supplemental Online Content: Nonauthor Collaborators
*First name, last name, and suffix (if applicable) are required and will appear in PubMed.

***First Name and Middle Initial(s)**	***Last Name**	***Suffix (eg, Jr, III)**	Academic Degrees	Institution	Location (city, state/province, country)	Role or Contribution, eg, chair, principal investigator	Group (if more than 1 Group listed in the byline) and/or Subgroup (eg, Steering Committee)
Nathan	Boucher		PhD	VA Durham Healthcare System	Durham, NC	Site Collaborator	CORC Participating Collaborators
Nicholas	Burwick		MD	VA Puget Sound Healthcare System	Seattle, WA	Physician Consultant	CORC Participating Collaborators
Aissa	Cabrales		BA	VA Ann Arbor Healthcare System	Ann Arbor, MI	Research Assistant	CORC Participating Collaborators
Jennifer	Cano		MPH	VA Ann Arbor Healthcare System	Ann Arbor, MI	Data Analyst	CORC Participating Collaborators
Wen	Chai		BS	VA Durham	Durham, NC	Data Analyst	CORC Participating Collaborators
Jason	Chen		MD	VA Portland Healthcare System	Portland, OR	Site Collaborator	CORC Participating Collaborators
Kei-Hoi	Cheung		PhD	VA Connecticut Healthcare System	West Haven, CT	Site Collaborator	CORC Participating Collaborators
Kristina	Crothers		MD	VA Puget Sound Healthcare System	Seattle, WA	Site Collaborator	CORC Participating Collaborators
Jeffrey	Curtis		MD	VA Ann Arbor Healthcare System	Ann Arbor, MI	Physician Consultant	CORC Participating Collaborators
Marie	Davis		MD	VA Puget Sound Healthcare System	Seattle, WA	Physician Consultant	CORC Participating Collaborators
Emily	Del Monico		MPH	VA Ann Arbor Healthcare System	Ann Arbor, MI	Research Assistant	CORC Participating Collaborators
Aram	Dobalian		PhD, JD	VA Greater Los Angeles Healthcare System	Los Angeles, CA	Physician Consultant	CORC Participating Collaborators
Jacob	Doll		MD	VA Puget Sound Healthcare System	Seattle, WA	Physician Consultant	CORC Participating Collaborators
Jason	Dominitz		MD	VA Puget Sound Healthcare System	Seattle, WA	Physician Consultant	CORC Participating Collaborators
McKenna	Eastment		MD	VA Puget Sound Healthcare System	Seattle, WA	Physician Consultant	CORC Participating Collaborators

Supplemental Online Content: Nonauthor Collaborators
*First name, last name, and suffix (if applicable) are required and will appear in PubMed.

***First Name and Middle Initial(s)**	***Last Name**	***Suffix (eg, Jr, III)**	Academic Degrees	Institution	Location (city, state/province, country)	Role or Contribution, eg, chair, principal investigator	Group (if more than 1 Group listed in the byline) and/or Subgroup (eg, Steering Committee)
Vincent	Fan		MD	VA Puget Sound Healthcare System	Seattle, WA	Physician Consultant	CORC Participating Collaborators
Jacqueline	Ferguson		PhD	VA Palo Alto Healthcare System	Palo Alto, CA	Site Collaborator	CORC Participating Collaborators
Breanna	Floyd		MPH	VA Durham Healthcare System	Durham, NC	Site Collaborator	CORC Participating Collaborators
Alexandra	Fox		MS	VA Puget Sound Healthcare System	Seattle, WA	Data Analyst	CORC Participating Collaborators
Matthew	Goetz		MD	VA Greater Los Angeles Healthcare System	Los Angeles, CA	Physician Consultant	CORC Participating Collaborators
Diana	Govier		PhD	VA Portland Healthcare System	Portland, OR	Data Analyst	CORC Participating Collaborators
Pamela	Green		PhD	VA Puget Sound Healthcare System	Seattle, WA	Data Manager	CORC Participating Collaborators
Susan Nicole	Hastings		MD	VA Durham Healthcare System	Durham, NC	Physician Consultant	CORC Participating Collaborators
Katie	Hauschildt		PhD	VA Ann Arbor Healthcare System	Ann Arbor, MI	Site Collaborator	CORC Participating Collaborators
Eric	Hawkins		PhD	VA Puget Sound Healthcare System	Seattle, WA	Site Collaborator	CORC Participating Collaborators
Paul	Hebert		PhD	VA Puget Sound Healthcare System	Seattle, WA	Site Collaborator	CORC Participating Collaborators
Mark	Helfand		PhD	VA Portland Healthcare System	Portland, OR	Site Collaborator	CORC Participating Collaborators
Alex	Hickok		MS	VA Portland Healthcare System	Portland, OR	Data Analyst	CORC Participating Collaborators
Dana	Horowitz		MSW	VA Ann Arbor Healthcare System	Ann Arbor, MI	Research Assistant	CORC Participating Collaborators
Catherine	Hough		MD	VA Portland Healthcare System	Portland, OR	Physician Consultant	CORC Participating Collaborators

Supplemental Online Content: Nonauthor Collaborators
*First name, last name, and suffix (if applicable) are required and will appear in PubMed.

***First Name and Middle Initial(s)**	***Last Name**	***Suffix (eg, Jr, III)**	Academic Degrees	Institution	Location (city, state/province, country)	Role or Contribution, eg, chair, principal investigator	Group (if more than 1 Group listed in the byline) and/or Subgroup (eg, Steering Committee)
Elaine	Hu		MS	VA Puget Sound Healthcare System	Seattle, WA	Data Analyst	CORC Participating Collaborators
Kevin	Ikuta		MD	VA Greater Los Angeles Healthcare System	Los Angeles, CA	Physician Consultant	CORC Participating Collaborators
Barbara	Jones		MD	VA Salt Lake City Healthcare System	Salt Lake City, UT	Site Collaborator	CORC Participating Collaborators
Makoto	Jones		MD	VA SLC Healthcare System	Salt Lake City, UT	Physician Consultant	CORC Participating Collaborators
Lee	Kamphuis		MPH	VA Ann Arbor Healthcare System	Ann Arbor, MI	Project Manager	CORC Participating Collaborators
Brystana	Kaufman		PhD	VA Durham Healthcare System	Durham, NC	Site Collaborator	CORC Participating Collaborators
Sara	Knight		PhD	VA Salt Lake City Healthcare System	Salt Lake City, UT	Site Collaborator	CORC Participating Collaborators
Anna	Korpak		PhD	VA Puget Sound Healthcare System	Seattle, WA	Data Analyst	CORC Participating Collaborators
Peggy	Korpela		MPH	VA Ann Arbor Healthcare System	Ann Arbor, MI	Research Assistant	CORC Participating Collaborators
Kyle	Kumbier		MS	VA Ann Arbor Healthcare System	Ann Arbor, MI	Data Analyst	CORC Participating Collaborators
Kenneth	Langa		MD	VA Ann Arbor Healthcare System	Ann Arbor, MI	Physician Consultant	CORC Participating Collaborators
Ryan	Laundry		BS	VA Puget Sound Healthcare System	Seattle, WA	Data Analyst	CORC Participating Collaborators
Stacy	Lavin		PhD	VA Durham Healthcare System	Durham, NC	Site Collaborator	CORC Participating Collaborators
Yuli	Li		MS	VA Connecticut Healthcare System	West Haven, CT	Site Collaborator	CORC Participating Collaborators
Jennifer	Linquist		PhD	VA Durham Healthcare System	Durham, NC	Data Analyst	CORC Participating Collaborators

Supplemental Online Content: Nonauthor Collaborators
*First name, last name, and suffix (if applicable) are required and will appear in PubMed.

***First Name and Middle Initial(s)**	***Last Name**	***Suffix (eg, Jr, III)**	Academic Degrees	Institution	Location (city, state/province, country)	Role or Contribution, eg, chair, principal investigator	Group (if more than 1 Group listed in the byline) and/or Subgroup (eg, Steering Committee)
Holly	McCready		BS	VA Portland Healthcare System	Portland, OR	Project Manager	CORC Participating Collaborators
Martha	Michel		PhD	VA Puget Sound Healthcare System	Seattle, WA	Data Analyst	CORC Participating Collaborators
Amy	Miles		MPH	VA Durham Healthcare System	Durham, NC	Site Collaborator	CORC Participating Collaborators
Jessie	Milne		MPH	VA Ann Arbor Healthcare System	Ann Arbor, MI	Research Assistant	CORC Participating Collaborators
Max	Monahan		MPH	VA Ann Arbor Healthcare System	Ann Arbor, MI	Project Manager	CORC Participating Collaborators
Daniel	Morelli		BA	VA Puget Sound Healthcare System	Seattle, WA	Data Analyst	CORC Participating Collaborators
Pradeep	Mutalik		MD	VA Connecticut Healthcare System	West Haven, CT	Site Collaborator	CORC Participating Collaborators
Jennifer	Naylor		MD	VA Durham Healthcare System	Durham, NC	Site Collaborator	CORC Participating Collaborators
Meike	Neiderhausen		PhD	VA Portland Healthcare System	Portland, OR	Data Analyst	CORC Participating Collaborators
Summer	Newell		PhD	VA Portland Healthcare System	Portland, OR	Site Collaborator	CORC Participating Collaborators
Shannon	Nugent		PhD	VA Portland Healthcare System	Portland, OR	Site Collaborator	CORC Participating Collaborators
Michael	Ong		MD, PhD	VA Greater Los Angeles Healthcare System	Los Angeles, CA	Physician Consultant	CORC Participating Collaborators
Thomas	Osborne		MD	VA Palo Alto Healthcare System	Palo Alto, CA	Site Collaborator	CORC Participating Collaborators
Matthew	Peterson		MS	VA Portland Healthcare System	Portland, OR	Data Manager	CORC Participating Collaborators
Alexander	Peterson		MS	VA Puget Sound Healthcare System	Seattle, WA	Data Analyst	CORC Participating Collaborators

Supplemental Online Content: Nonauthor Collaborators
*First name, last name, and suffix (if applicable) are required and will appear in PubMed.

***First Name and Middle Initial(s)**	***Last Name**	***Suffix (eg, Jr, III)**	Academic Degrees	Institution	Location (city, state/province, country)	Role or Contribution, eg, chair, principal investigator	Group (if more than 1 Group listed in the byline) and/or Subgroup (eg, Steering Committee)
Hallie	Prescott		MD	VA Ann Arbor Healthcare System	Ann Arbor, MI	Physician Consultant	CORC Participating Collaborators
Nallakkandi	Rajeevan		PhD	VA Connecticut Healthcare System	West Haven, CT	Site Collaborator	CORC Participating Collaborators
Ashok	Reddy		MD	VA Puget Sound Healthcare System	Seattle, WA	Physician Consultant	CORC Participating Collaborators
Marylena	Rouse		BS	VA Ann Arbor Healthcare System	Ann Arbor, MI	Research Assistant	CORC Participating Collaborators
Mazhgan	Rowneki		MPH	VA Portland Healthcare System	Portland, OR	Data Manager	CORC Participating Collaborators
Som	Saha		MD	VA Portland Healthcare System	Portland, OR	Physician Consultant	CORC Participating Collaborators
Sameer	Saini		MD	VA Ann Arbor Healthcare System	Ann Arbor, MI	Physician Consultant	CORC Participating Collaborators
Javeed	Shah		MD	VA Puget Sound Healthcare System	Seattle, WA	Physician Consultant	CORC Participating Collaborators
Troy	Shahoumian		PhD	VA Palo Alto Healthcare System	Palo Alto, CA	Data Analyst	CORC Participating Collaborators
Aasma	Shaukat		MD	VA Minneapolis Healthcare System	Minneapolis, MN	Physician Consultant	CORC Participating Collaborators
Megan	Shepherd-Banigan		PhD	VA Durham Healthcare System	Durham, NC	Site Collaborator	CORC Participating Collaborators
Whitney	Showalter		PhD	VA Puget Sound Healthcare System	Seattle, WA	Project Manager	CORC Participating Collaborators
Christopher	Slatore		MD	VA Portland Healthcare System	Portland, OR	Physician Consultant	CORC Participating Collaborators
Nicholas	Smith		PhD	VA Puget Sound Healthcare System	Seattle, WA	Site Collaborator	CORC Participating Collaborators
Battista	Smith		MPH	VA Durham Healthcare System	Durham, NC	Project Manager	CORC Participating Collaborators

Supplemental Online Content: Nonauthor Collaborators
*First name, last name, and suffix (if applicable) are required and will appear in PubMed.

***First Name and Middle Initial(s)**	***Last Name**	***Suffix (eg, Jr, III)**	Academic Degrees	Institution	Location (city, state/province, country)	Role or Contribution, eg, chair, principal investigator	Group (if more than 1 Group listed in the byline) and/or Subgroup (eg, Steering Committee)
Pradeep	Suri		MD	VA Puget Sound Healthcare System	Seattle, WA	Physician Consultant	CORC Participating Collaborators
Jeremy	Sussman		MD	VA Ann Arbor Healthcare System	Ann Arbor, MI	Physician Consultant	CORC Participating Collaborators
Yumie	Takata		PhD	VA Portland Healthcare System	Portland, OR	Site Collaborator	CORC Participating Collaborators
Alan	Teo		MD	VA Portland Healthcare System	Portland, OR	Site Collaborator	CORC Participating Collaborators
Eva	Thomas		MPH	VA Puget Sound Healthcare System	Seattle, WA	Data Analyst	CORC Participating Collaborators
Laura	Thomas		MPH/MSW	VA Ann Arbor Healthcare System	Ann Arbor, MI	Project Manager	CORC Participating Collaborators
Anais	Tuepker		PhD	VA Portland Healthcare System	Portland, OR	Site Collaborator	CORC Participating Collaborators
Zachary	Veigulis		MS	VA Palo Alto Healthcare System	Palo Alto, CA	Data Analyst	CORC Participating Collaborators
Elizabeth	Vig		MD	VA Puget Sound Healthcare System	Seattle, WA	Site Collaborator	CORC Participating Collaborators
Kelly	Vranas		MD	VA Portland Healthcare System	Portland, OR	Site Collaborator	CORC Participating Collaborators
Xiao Qing	Wang		MPH	VA Ann Arbor Healthcare System	Ann Arbor, MI	Data Manager	CORC Participating Collaborators
Katrina	Wicks		MPH	VA Puget Sound Healthcare System	Seattle, WA	Data Analyst	CORC Participating Collaborators
Kara	Winchell		MA	VA Portland Healthcare System	Portland, OR	Project Manager	CORC Participating Collaborators
Edwin	Wong		PhD	VA Puget Sound Healthcare System	Seattle, WA	Site Collaborator	CORC Participating Collaborators
Chris	Woods		MD	VA Durham Healthcare System	Durham, NC	Physician Consultant	CORC Participating Collaborators

Supplemental Online Content: Nonauthor Collaborators
*First name, last name, and suffix (if applicable) are required and will appear in PubMed.

***First Name and Middle Initial(s)**	***Last Name**	***Suffix (eg, Jr, III)**	Academic Degrees	Institution	Location (city, state/province, country)	Role or Contribution, eg, chair, principal investigator	Group (if more than 1 Group listed in the byline) and/or Subgroup (eg, Steering Committee)
Katherine	Wysham		MD	VA Puget Sound Healthcare System	Seattle, WA	Physician Consultant	CORC Participating Collaborators
Lei	Yan		PhD	VA Connecticut Healthcare System	West Haven, CT	Site Collaborator	CORC Participating Collaborators
Donna	Zulman		MD	VA Palo Alto Healthcare System	Palo Alto, CA	Physician Consultant	CORC Participating Collaborators
