# Supplementary material for: Self-Reported Everyday Functioning After COVID-19 Infection
Source: JAMA Netw Open. 2024 Mar 1;7(3):e240869. doi: 10.1001/jamanetworkopen.2024.0869 (PMC10907923; doi:10.1001/jamanetworkopen.2024.0869)
Supplement: Supplement 3. — Data Sharing Statement [file jamanetwopen-e240869-s003.pdf]

Data Sharing Statement

Data
Data available: Yes
Data types: Deidentified participant data
How to access data: Data will be made available from the VA/VINCI data repository on completion of a valid Department of Veterans Affairs Data Use Agreement
When available: With publication

Supporting Documents
Document types: Statistical/analytic code
How to access documents: It has been uploaded into submission portfolio
When available: With publication

Additional Information
Who can access the data: Automatically
Types of analyses: Any purpose approved for use by a valid Department of Veterans Affairs Data Use Agreement
Mechanisms of data availability: standard valid Department of Veterans Affairs Data Use Agreement process
